# Molecular-Level Interactions between Engineered Materials and Cells

**DOI:** 10.3390/ijms20174142

**Published:** 2019-08-25

**Authors:** Yoon-ha Jang, Xuelin Jin, Prabakaran Shankar, Jung Heon Lee, Kyubong Jo, Kwang-il Lim

**Affiliations:** 1 Department of Chemical and Biological Engineering, Sookmyung Women’s University, Seoul 04310, Korea; 2 Department of Chemistry and Integrated Biotechnology, Sogang University, Seoul 04107, Korea; 3 School of Advanced Materials Science and Engineering, Sungkyunkwan University (SKKU), Suwon 16419, Korea

**Keywords:** mechanotransduction, materials engineering, cell surface sensors, genome states, cellular responses

## Abstract

Various recent experimental observations indicate that growing cells on engineered materials can alter their physiology, function, and fate. This finding suggests that better molecular-level understanding of the interactions between cells and materials may guide the design and construction of sophisticated artificial substrates, potentially enabling control of cells for use in various biomedical applications. In this review, we introduce recent research results that shed light on molecular events and mechanisms involved in the interactions between cells and materials. We discuss the development of materials with distinct physical, chemical, and biological features, cellular sensing of the engineered materials, transfer of the sensing information to the cell nucleus, subsequent changes in physical and chemical states of genomic DNA, and finally the resulting cellular behavior changes. Ongoing efforts to advance materials engineering and the cell–material interface will eventually expand the cell-based applications in therapies and tissue regenerations.

## 1. Introduction

Biological molecules, including DNA, RNA, and proteins, are introduced into mammalian cells to control the physiology, function, behavior, and fate of the cells [1,2,3]. These molecular treatments lead to the expression of the external genes or changes in endogenous gene expression. These genetic manipulations subsequently alter interactions between cellular molecules involved in the targeted cellular pathways, eventually enabling the control of mammalian cells. However, the introduced biological molecules may also eventually alter other cellular processes that were not targeted, via cross-talking with multiple intracellular pathways at the same time or unspecific and random interactions with cellular components, thereby causing unwanted side effects [4,5,6]. In addition, this conventional method may be less than ideal in realizing the intended cellular state.

Recent attempts to improve the control of mammalian cells have explored inducing cellular responses to materials [7,8,9]. This new approach may eliminate the need to introduce biological molecules into cells or enable better control of mammalian cells when combined with currently used biological methods. In the present review, we introduce multiple studies concerning material science and engineering applications to control cells. This review covers the construction of diverse forms of materials with varied physical and chemical characteristics such as stiffness, viscosity, morphology, and chemical structure, molecular mechanisms underlying cellular sensing of engineered materials and transfer of the sensing information to the cell nucleus, the changes in the physical and chemical states of genomic DNA, and the subsequent alterations in endogenous gene expression.

## 2. Engineered Materials

Cellular interactions with the external environment are multiscale phenomena from atomic and molecular levels to the micrometer-scale. This means that materials used as artificial external environments to control the physiology, function, behavior, and fate of cells need to be engineered to have certain layers, patterns, or structures with sizes ranging from a few nanometers to micrometers [10,11]. Physical characteristics of the engineered environments include stiffness, morphology, and elasticity. Chemical characteristics include hydrophilicity and hydrophobicity and involve chemical moieties that may significantly affect cellular pathways and processes [12,13]. Various materials of different forms and chemical compositions, including polymer/inorganic thin films, composite scaffolds, hydrogels, and fibrous scaffolds, have been constructed as cell culture substrates to control mammalian cells. In this section, we discuss materials that have been recently constructed for biomedical applications and their physical, mechanical, and chemical properties.

### 2.1. Physical/Mechanical Properties of Materials—Stiffness and Viscosity

Cellular responses to external forces provided by materials can initiate or alter intracellular signaling pathways, ultimately leading to changes in proliferation, development, and differentiation of cells [14,15,16]. The impact of extrinsic or intrinsic mechanical forces on a cell can be understood by using certain materials engineered to have desired physical or mechanical properties, which include shape, strength, elasticity, stiffness, viscosity, and topography. These properties may exert unique effects on different cells [17].

Soft materials are extensively used to study the effect of physical and mechanical properties of materials on cellular behaviors, because of the ease in manufacturing the materials with variable structures, surface morphologies, and stiffness. Generally, natural gels (gelatin, collagen, Matrigel, and fibrin) have excellent biocompatibility but have poor tunability for physical and chemical properties. For example, it is quite difficult to tune the stiffness of pristine gelatin, which is approximately 10 kPa, unless a significant amount of crosslinking agents or nanowires are incorporated extrinsically [18,19]. Conversely, it is quite easy to change the physical and mechanical properties of synthetic hydrogels, such as polyacrylamide (PAAm) [20], polyethylene glycol (PEG) [21,22], polycaprolactone (PCL) [23], and polydimethylsiloxane (PDMS) [24,25,26], simply by changing the preparation conditions. For example, PAAm gels were prepared to have different stiffness of around 8 kPa and 100 kPa by varying the amount of precursors, acrylamide and bis-acrylamide, as 6% and 25%, respectively [20].

The effects of substrate stiffness on the biophysical regulation of cell behavior have been the main focus of many studies (Table 1) [27]. The stiffness of PAAm gels has been controlled by changing their thickness, coating their surface with extracellular matrix (ECM) proteins such as collagens, forming a composite with nanoparticles, and modifying the preparation procedures that involve curing, micropatterning, and printing [11,14,28,29]. Fibronectin coated PAAm gels with varying stiffness of approximately 8.7 kPa and 113.2 kPa revealed different effects on NIH3T3 fibroblast cell focal adhesions, protein dynamics, and mechanosensing [20]. Comparisons with signaling proteins, such as focal adhesion kinase (FAK) and paxillin, have revealed that the mobilities of structural proteins, including tensin, talin, and vinculin, on the cell surface are significantly decreased with increasing substrate stiffness, which eventually alters the cellular mechanosensing and mechano-response of NIH3T3 cells. Similarly, collagen-coated PAAm substrate with stiffness of approximately 5.7 kPa could significantly enhance the nuclear translocation of transcription factors through mechanical forces compared to a substrate with lower stiffness of approximately 150 Pa [30].

In addition to matrix stiffness, surface viscosity-driven ligand mobility change significantly affects the cellular behavior like actin flows, adhesion growth, Yes-associated protein (YAP) nuclear translocation, and myoblast differentiation [31]. The lipid bilayers of 1,2-dioleoyl-sn-glycero-3-phosphocholine (DOPC) fluid and 1,2-dipalmitoyl-sn-glycero-3-phosphocholine (DPPC) gel were used to construct substrates with different viscosities to understand the cellular behavior of C2C12 mouse myoblasts [31]. DPPC substrates with high viscosity generated a cellular response and hindered flow of actin via the strong adsorption of the lipid membrane. In contrast, DOPC substrates with lower viscosity conferred low resistance for the cell membrane, which enhanced actin flow [31].

Studies mentioned in this subsection show that the viscoelastic properties (defined with viscosity and stiffness) of substrate materials can significantly affect cellular processes and behaviors. Since each cell type may have characteristic interactions with materials, it is important to carefully consider the cell type when designing the materials to obtain desired cellular responses.

### 2.2. Physical/Mechanical Properties of Materials—Geometrical Factors Including Shape and Morphology

**Figure 1:**
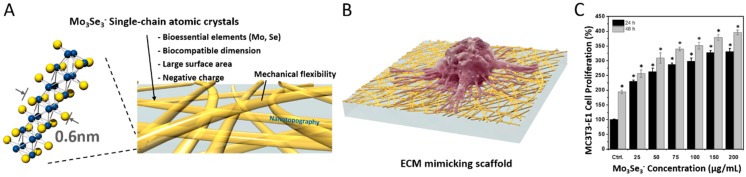
Mo_3_Se_3_^−^ SCAC-based extracellular matrix (ECM) mimicking materials [34]. (**A**) Properties of Mo_3_Se_3_^−^ SCACs to mimic ECM. (**B**) ECM-mimicking scaffold prepared by spray coating of Mo_3_Se_3_^−^ SCACs onto a glass substrate. (**C**) Proliferation of MC3T3-E1 cell lines treated with media containing different concentrations of Mo_3_Se_3_^−^ SCACs (* for *p* < 0.0001). All statistical analysis was performed with the control data. “Reprinted with permission from ref. [34]. Copyright 2018, American Chemical Society.”

Materials of different shape or morphology, exemplified by spherical, triangular, and square surfaces of varying sizes or one, two, and three dimensional structures, produced distinct results in inducing cellular responses [11,15,32,33]. The surface to volume ratio, surface area, and shape or morphology of materials affect cellular responses, often recognized by changes in cell shape, spreading, and mobility. Inorganic nanomaterials are promising candidates for fabrication of substrates with different dimensions. A recent study with a one-dimensional (1D) inorganic nanomaterial (molybdenum selenide, Mo_3_Se_3_^−^) affirmed the enhanced cellular response to physical properties of materials, such as dimension comparable to ECM molecules, mechanical flexibility, and topography [34]. The authors reported high cell proliferation on 1D Mo_3_Se_3_^−^ single chain atomic crystals (SCACs) (Figure 1). The authors reported significant increases of proliferation of L-929 fibroblast and MC3T3-E1 osteoblast cell lines up to 268.4 ± 24.4% and 396.2 ± 8.1%, respectively, at 48 h post culturing with Mo_3_Se_3_^−^ SCACs (Figure 1). 1D Mo_3_Se_3_^−^ SCAC nanowire with negative surface charge, mechanical flexibility, large surface area, and small diameter (approximately 0.6 nm) mimicked ECM and improved the cellular response [34]. Furthermore, others prepared gold (Au) nanomaterials of different sizes and shapes (sphere, star, and nanorod) and demonstrated the effects of the materials on osteogenic differentiation of human mesenchymal stem cells (hMSC) and resulting alkaline phosphatase (ALP) activity and calcium deposition [35]. The effects of materials’ size and shape-dependent factors could be extended to alter nuclear signaling pathways. An investigation of the interaction of Au nanostars with the nuclei of cancer cells revealed major changes in the nuclear state [36].

Geometrical factors can also influence cell proliferation, cell-generated traction forces, and other cellular functions [37]. In general, studies on the effects of geometrical factors on cell interactions have mainly used polymer hydrogels, polymer casted substrates, electrospun fibrous scaffolds, and nanocrystalline substrates [38,39]. The micropatterning technique has been actively utilized to develop desired patterns or geometries on soft and hard materials. Cross-linking, cleavage of hydrogen bonds, and hydration process along with stamping can be useful in constructing hydrogels with controlled geometry [39]. For example, a study employing soft PAAm hydrogel substrates with defined geometries has provided a great deal of information concerning human mammary epithelial (MCF-10A) cells’ behavior on symmetric and asymmetric geometries [40]. Both soft (1 kPa) and stiff (7 kPa) PAAm gels with an identical surface area of 2500 μm^2^ but with different surface shapes (square, triangular, and rectangular; aspect ratio: 1:1, 1:1, and 1:4, respectively) were developed to investigate the geometric effects of materials on cellular interactions. The results indicated that cell-generated traction forces for protrusion, adhesion, and spreading mainly depended on the shapes of the ECM matrix, irrespective of material stiffness.

**Figure 2:**
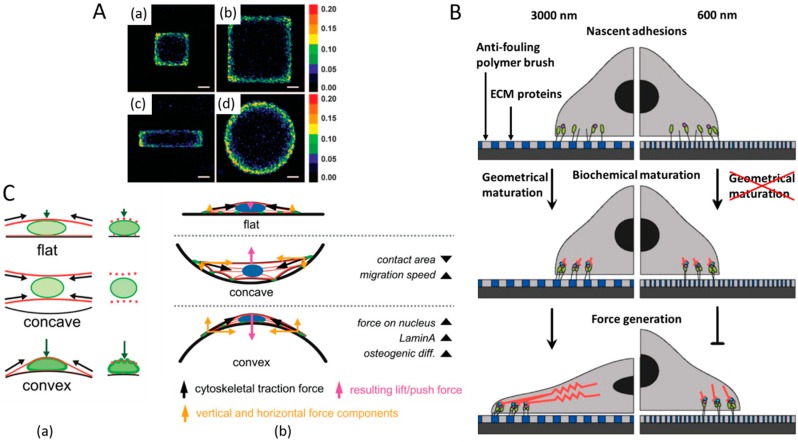
The effects of substrate geometry on cells. (**A**) The patterns of forces exerted by the cells responding to the edges and boundaries of different substrates. (a) Colorimetric stacked images of cell proliferation in a small (250 µm edge) square, (b) large (500 µm edge) square, (c) small (125 × 500 µm) rectangular, and (d) large (564 µm diameter) circular islands [37]. “Reprinted with permission from ref. [37]. Copyright 2005, National Academy of Sciences” (**B**) A model of geometrical, biochemical, and mechanical maturation of integrin-mediated cell adhesion and behaviour after responding to nanopatterned matrices [42]. “Reprinted with permission from [42]. Copyright 2014, American Chemical Society.” (**C**) Schematic representation of (a) the cytoskeletal forces acting on the nucleus (F-actin in red and lamin-A in green) and (b) the proposed geometry-induced changes in cellular attachment and forces on the nucleus for flat, concave and convex surfaces [43]. “Reprinted with permission from ref. [43]. Reproduced with permission under Creative Commons Attribution 4.0 International License http://creativecommons.org/licenses/by/4.0/.

Especially, the colloidal lithography technique can be used to develop nanopatterned substrates decorated with Au nanoparticles. Au nanoparticles can be easily functionalized with chemical or biological moieties [10,41]. For example, Nelson et al. used fibronectin coated Au islands with square, rectangular, and spherical geometries to assess the response of cells to the geometry of the substrate [37]. The pattern of forces exerted by the cells corresponded to the edges and boundaries of the substrate (Figure 2A). Likewise, a study demonstrated force-dependent focal adhesion of cells using Au substrates patterned in different sizes (0.1, 0.6, and 3.0 μm). The study reported constraints in localization and adhesion dynamics of cells, which determined cell fates by the geometrical patterns of the materials, independent of matrix stiffness (Figure 2B) [42]. The collective findings indicate that both soft hydrogels and metal-based micropatterned substrates with different shapes and geometries can be used to explore the mechanotransduction mechanism for the regulation of cells.

The impact of two-dimensional (2D) geometrical substrates on cells has been also studied. Although 2D substrates may be suitable to investigate the influence of individual geometrical factors on cellular activities, three-dimensional (3D) geometrical substrates that more realistically support cell growth and interactions with their surroundings can be more useful, as they can closely mimic the cellular environment in vivo. A few studies have explored the influence of 3D geometrical factors on cell behaviors. Werner et al. developed poly(trimethylene carbonate)-based 3D microtopographic cell culture chips comprising concave and convex spherical structures with diameter of 250 μm and principal curvature of 1/125 μm^−1^ using stereolithography (Figure 2C) [43]. The authors described that the contact area on 3D concave structure increased the migration speed of MSCs, while the 3D convex structure induced osteogenic differentiation of the cells by activating their cytoskeletal forces on the nucleus. Cytoskeleton-tension-associated pulling or pushing force was produced responding to the concave or convex 3D structure, respectively, significantly influencing the cell attachment on the surface and causing nuclear deformation as well.

Another study using a quasi-2D fibrous nanopattern (developed through electrospun nanofiber lithography technique) also indicated the importance of the 3D geometry on cell responses [44]. The poly (methyl methacrylate) (PMMA) nanofibers with various diameters (250–1000 nm) developed on prefunctionalized silane initiator substrate acted as a barrier blocking protein adsorption. This structure was obtained through the growth of a polymer (oligo(ethylene glycol methacrylate)) brush, which is protein- and cell-resistant, and removal of PMMA using an electrospun nanofiber lithography technique. The size of the fibrous fibronectin structure turned out to effectively influence the HaCaT cell adhesion, spreading, and shaping. In another study, electrodeposited Au nanospikes with a 3D leaf-like structure promoted MSC alignment and neurogenic differentiation [45].

Extensive investigation with various surface patterns, including grooves, pits, pillars, and ridges, has highlighted the impact of nanopatterns of supporting materials on cell functions. The materials with these topographic patterns act as anchoring sites for the adhesion of proteins present in the cell membranes and induce changes in cell size and shape, differentiation, and physiological phenotype [10,11,27,46,47]. Among several polymer-based materials, PDMS, PMMA, and poly(lactide-co-glycolide) (PLGA) were used to prepare distinct surface patterns with varying degrees of stiffness to understand the mechanisms of cellular interactions with materials [48]. The use of fibronectin-coated PDMS substrates that were engineered with anisotropic nanogratings and isotropic nanopillars of different dimensions helped to find the effects of nanotopographies on normal human lung fibroblasts (NHLFs) [49]. The results suggested that nanoscale gratings can better facilitate the formation and growth of sites of focal adhesion compared with nanopillars. Importantly, the observation that gratings with a certain dimension caused nuclear deformation, suggested that appropriate patterns could facilitate nuclear mechanosensing.

Furthermore, nanotopography generated by layering nano-sized silica beads could alter the state of genomic DNA of mammalian cells, ultimately affecting retroviral integration patterns. Cells on the bead layer with the highest curvature often harbored the retrovirus genomes in the regulatory domains of their nuclear DNA during infection [9]. This new finding suggests that making cells respond to engineered substrate can be a way to control cellular responses to invading viruses.

Another study demonstrated the need of rational design of specific topographic patterns for certain cell lines. For example, poly(urethane acrylate) (PUA) nanoposts with an approximate thickness of 50 μm and gradient density specifically acted on melanoma cells [7]. Pitrez et al. demonstrated that careful optimization of the topography is important to control cell fate. The authors reported that cell aging was also affected by topography of substrate [50]. A PDMS substrate with a height of 1.5 μm and width of 5 μm, comprising grooves and ridges, induced more extensive disruption between the cytoskeleton and nucleoskeleton, and increased the activity of DNA-dependent protein kinase, compared with other dimensions and a flat substrate [50].

Metal-based substrates with certain topographies have also been used for other biomedical applications [51,52,53]. For example, titanium (Ti) nanostructured surfaces are important nanomaterials for dental and orthopedic implantations, enabling the enhanced osseointegration and soft tissue integration due to the synergistic effects of their nanotopographical and mechanical properties [51,54,55,56,57]. A Ti surface with optimal nanotopographic pattern, wettability, and mechanical strength could effectively induce mechanotransduction [56]. Human gingival fibroblast (hGF) cells showed enhanced cell attachment, proliferation, and differentiation on Ti surfaces with 74 nm diameter pores, surface roughness of 41.6 nm, surface area of 30.4 μm^2^, and hydrophilicity of 65.5°.

The geometrical factors of materials, such as size, shape, dimension, topography, and surface area play important roles in changing or controlling behaviors of cells, because the patterns of forces exerted by cells are significantly affected by those factors (Table 2). Thus, better design and engineering of these physical factors of materials would be one of the keys to obtain desired states and functions of cells.

### 2.3. Physical/Mechanical Properties of Materials—Electrical and Magnetic Potentials

Surface charge of materials can also act as a stimulus and induce mechanotransduction of cells (Table 3). Electric and magnetic fields can be desirable responsive platforms to control cell fate, as these external factors directly affect the cell–material interaction mechanisms [58]. Electrical potentials are generally activated by applying electric fields through electrodes deposited on electroconductive materials, while magnetic fields can be activated on magnetic responsive materials by magnets or electromagnetic induction coils [58].

Wei et al. demonstrated that an electric potential can be applied directly on material to guide the cellular response, irrespective of stiffness and chemical inducers [59]. A surface potential sensitive polypyrrole (Ppy) array on Ti surface could induce or affect the intracellular mechanotransduction and cytoskeleton organization when MSCs were attached to the surface and detached from the surface. With the application of surface potential, the Ti surface was transformed to highly adhesive hydrophobic nanotubes and poorly adhesive hydrophilic nanotips during the oxidation and reduction potential cycle, respectively. The dynamic switching of nanotube/nanotip regulated the cell–material interactions, also guided by the surface properties of materials such as wettability, adhesive force, and elastic modulus. The stimulus-responsive materials induced osteogenic differentiation of the cells in the cycle-dependent manner.

In other case, an external magnetic field was applied on a monolayer of RGD-functionalized magnetic nanoparticles (MNPs) conjugated on a glass substrate via a flexible and coiled poly(ethylene glycol) linker to investigate the effect of the tether mobility of RGD on cell adhesion and spreading [60]. RGD mobility is known to directly influence the formation of focal adhesion and mechanotransduction of cells. In the absence of magnetic field, high RGD tether mobility delayed adhesion and spreading of hMSCs. Once magnetic field was applied, RGD tether length decreased from ~72.2 nm to ~17.8 nm. This reduction of tether length significantly restricted the mobility of RGD, which finally resulted in enhanced adhesion and spreading, and mechanotransduction-mediated osteogenic differentiation of hMSCs.

A magnetic force pulse in the range of piconewtons can alter mechanical sensitive microenvironments. In this regard, magnetic nanomaterials were used to spatiotemporally manipulate mechanical force for a magneto-mechanotransduction mechanism [61]. The engineering of such magnetic nanomaterials can target the cell receptors and induce specific signaling pathways for cellular responses. The internalization of functionalized magnetic nanoparticles can affect the intracellular organelles and initiate cell apoptosis. Overall, the effect of magnetic fields has been widely studied for different applications, including regenerative medicine and cancer treatment. Two recently published reviews summarize the recent advances in understanding magnetic nanomaterials and their mechanotransduction effects on different cells [61,62].

Accompanying other physical stimuli, electric and magnetic fields can significantly alter the force-driven cell–material interactions. The design and construction of stimulus-responsive nanomaterials with characteristic stiffness, geometry, and topography would enable the control of cellular states and behaviors.

### 2.4. Chemical/Biological Functionalization of Materials

Chemical and biological functionalization is also useful to adjust cell–material interactions and to subsequently regulate cell metabolism. The interactions between cell receptors and chemical or biological moieties of material surface have been addressed [52,63]. Specific chemical functional groups or ECM molecules are generally chosen for the chemical or biological functionalization of material surfaces. On the surface, these molecules act as anchoring sites that interact directly with cell surface receptors [64].

**Figure 3:**
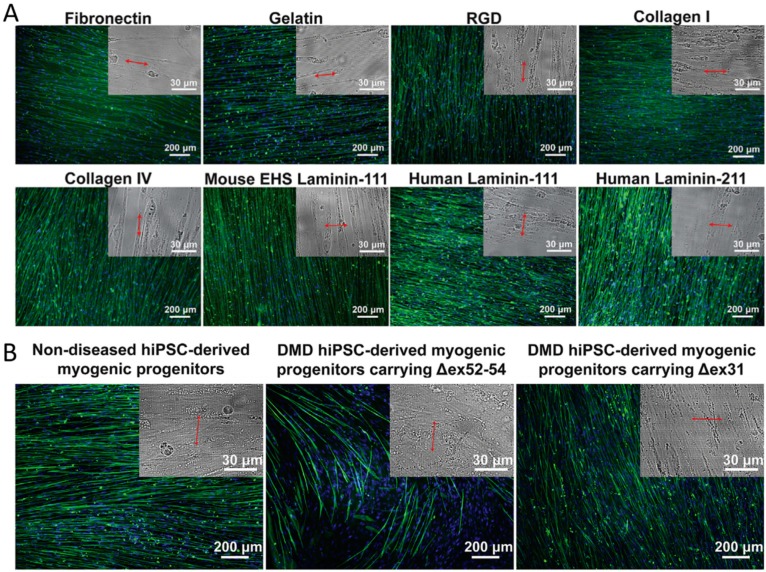
**Figure 3**. Myotube alignment and orientation on engineered substrates. (**A**) The effects of substrate-bound adhesion molecules on alignment and orientation of myotubes. Eight hundred nanometer grooved substrates were functionalized with different ECM components. (**B**) Myotube alignment and orientation on 800 nm grooved Matrigel-functionalized substrates to differentiate diseased and nondiseased cells (inset: bright-field images show nanogroove directions (marked with red arrows)) [65]. “Reprinted from ref. [65], Copyright 2018, with permission from Elsevier.”

ECM components, such as laminin, elastin, vitronectin, collagen, proteoglycans, and glycosaminoglycans, have unique physical and biological properties that facilitate cellular responses. A Matrigel-functionalized, 800 nm grooved PDMS substrates, have been used to distinguish diseased and nondiseased myotubes (Figure 3) [65]. The PDMS substrates patterned with 500, 800, 1000, 1500, and 3000 nm width parallel grooves (400 nm depth) were functionalized with Matrigel, gelatin, Arg-Gly-Asp (RGD) peptide, fibronectin, and type-I collagen to study the behavior of human embryonic stem cells (hESCs) derived myotubes. In the study, the myotubes were aligned in parallel on gelatin, RGD peptide, fibronectin, and type-I collagen functionalized 800 nm nanogrooved substrates, whereas they were aligned perpendicularly on the Matrigel-functionalized 800 nm nanogrooved substrate. Furthermore, the 800 nm substrates were functionalized with laminin-111, laminin-211, and type-IV collagen to identify the ECM component in Matrigel, enabling the cell–material interactions to align perpendicularly to the nanogrooves. Both laminin-111 and laminin-211 favored the perpendicular myotube alignment. This study exhibited that laminins could provide binding sites for α-dystroglycan on the surface of muscle cells through dystrophin-associated protein complex (DAPC)-mediated cytoskeleton–ECM linkage. It has been confirmed with heparin and anti-α-dystroglycan antibody IIH6 via disruption of the DAPC–laminin interactions. In another study, human corneal endothelial cells on different pillar structures functionalized with fibronectin–type-I collagen and laminin-chondroitin sulfate showed changes in gene expression [66]. The cells on the fibronectin–type-I collagen-coated micropillars exhibited high Na^+^/K^+^ ATPase and zonula occludens 1 (ZO-1) expression, leading to enhanced circularity.

Biomolecules such as proteins, nucleic acids, and polysaccharides contain amines (-NH_2_), carboxyl (-COOH), hydroxyl (-OH), methyl (-CH_3_), sulfhydryl (-SH), and phosphate (-PO_4_^−^) functional groups which play important roles in most molecular and cellular interactions. Thus, polymer-based cell-culturing substrates are functionalized with chemical groups such as amine, carboxylic, and hydroxyl to induce cell–material interactions [10,46,65,67,68,69,70]. For example, allylamine plasma polymer layer (PPAAm) coated on titanium (Ti) surface promoted adhesion of MG-63 osteoblastic cells [71]. Ti surface functionalized with positively charged PPAAm enhanced the osteoblastic cellular responses including focal contact formation and actin cytoskeleton development. Interestingly, the level of observed osteoblastic cellular responses on PPAAm-Ti surface was comparable to those of cells on type-I-collagen immobilized Ti surface. Similarly, the negatively charged pericellular hyaluronan (HA) promoted initial adhesion steps of osteoblast on the positively charged amino-functionalized Ti surface (PPAAm). Thus, the electrostatically controlled cell–material interaction aided cell adhesion, spreading, and associated behavior. In this regard, the polymer layer seems to mimic the function of collagen in the natural ECM [71].

In another case, Au surfaces functionalized with different chemical groups were used to investigate the surface chemistry-dependent integrin binding and signaling of osteoblast cells [72]. Self-assembled monolayers of alkanethiols like 1-dodecanethiol, 11-mercapto-1-undecanol, 11-mercaptoundecanoic acid, and 12-amino-1-mercaptododecane were used to modify the Au surface with different chemical groups, including CH_3_ (hydrophobic), OH (neutral and hydrophilic), COOH (negatively charged at pH 7.4), and NH_2_ (positively charged at pH 7.4), respectively. Among those, growing on OH- and NH_2_-terminated Au surfaces resulted in increases in expression of alkaline phosphatase, bone sialoprotein, and osteocalcin, activity of alkaline phosphatase, and matrix mineralization of MC3T3-E1 cells, likely due to the predominant binding of specific integrin receptors to the substrates. The selective binding of α_5_β_1_ and α_V_β_3_ integrin to functionalized surface affects the focal adhesion composition, osteoblast differentiation, signaling, and mineralization. The systematic investigation of the OH- and NH_2_-terminated substrates with β_1_ and β_3_ integrin blocking antibodies showed enhanced specific binding of β_1_ integrin which promoted the cellular activities. This result indicates that chemical modification of substrates could enhance the integrin binding specificity to activate specific signaling pathways and cellular behavior. Table 4 summarizes the effects of chemical and biological functionalization of culturing substrate on cells.

Engineering biomaterials to reach certain goals in biomedical applications begins with the right selection of organic, inorganic or, hybrid base materials with characteristic physio-chemical properties. Combining materials engineering with use of various physical, electrical/magnetic, and chemical external stimuli will further facilitate more sophisticated control of the states and behaviors of cells.

## 3. Cellular Sensing of Engineered Materials

In the process of cellular sensing of the surrounding environment, receptor proteins on the cell surface play major roles [73,74,75]. Cell surface receptors can recognize not only various natural ligands such as growth factors and hormones, but also diverse artificial features of engineered materials such as physical dimensions and chemical functional groups. In particular, physical factors are key targets for cells to recognize for sensing outer engineered materials. Mammalian cells use integrins, mechanosensitive ion channels, and G-protein coupled receptors (GPCRs) for this sensing process [73].

### 3.1. Sensing Receptors on the Cell Surface—Integrins

Integrin is a receptor protein whose extracellular head domain provides a site for ligand binding and its cytoplasmic tail domain interacts with intracellular molecules. The interactions involving integrins result in physical connections between extracellular and intracellular structural domains. Integrins recognize natural ECM components and various physical and biochemical cues from engineered materials ultimately causing changes in physiology, behaviors, and fate of cells. For example, integrin-mediated sensing of topographic features of the surrounding materials can affect the direction of cell migration. PUA mold materials with thickness of ~50 µm were used to construct an array of 600 nm diameter nanoposts with gradually varied spacing from 0.3 µm to 4.2 µm in one of the orthogonal directions and constant spacing in the other direction. On this material, melanoma cells migrated to the zones with a sparse density of the nanoposts. This is because the cells were able to more readily penetrate into the interpost space in sparser zones than in denser zones, and thus integrin-mediated adhesion to the substrate was increased. The cells migrated from denser to sparser zones where the cell–substrate contact was enhanced [7].

**Figure 4:**
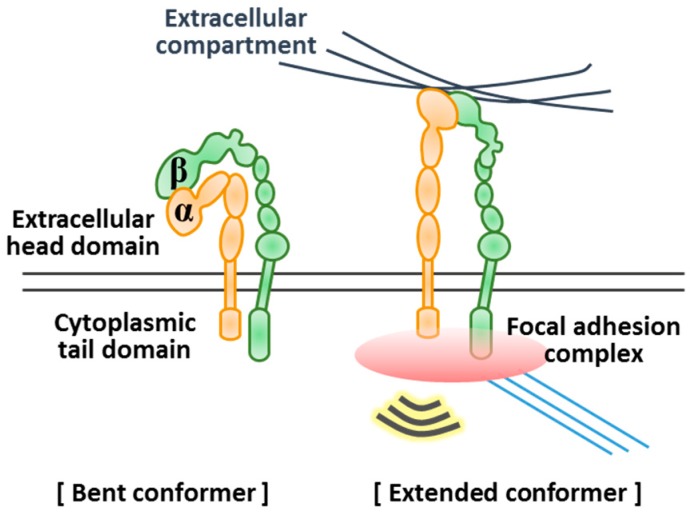
Conformational rearrangement of integrin upon attachment to substrate [79,80,81]. Integrin receptor proteins work as αβ heterodimers. When attaching to substrate, integrins undergo conformational rearrangements from bent conformation with low affinity for ligand to extended conformation with high affinity for ligand. This integrin binding to substrates induces signal transduction and provides physical connections between extracellular and intracellular regions through focal adhesion complex formation.

Integrin is a heterodimer composed of α and β subunits (Figure 4). Integrin can interact with various molecules [76]. There are 18 α and 8 β subunit genes in mammalian genomes, of which translation products can combine to form 24 different integrin proteins [76,77]. The extracellular domains of the α and β subunits contribute to form a ligand binding site and the resultant integrins selectively bind to different ligands [77]. As established examples of integrin–ECM component interactions, all five α_v_ integrins (α_v_β_1_, α_v_β_3_, α_v_β_5_, α_v_β_6_, and α_v_β_8_), two β_1_ integrins (α_5_β_1_ and α_8_β_1_), and α_IIb_β_3_ bind to the ligands containing RGD sequences, such as fibronectin, vitronectin, and laminin. Additionally, a set of β_1_ integrins (α_1_β_1_, α_2_β_1_, α_10_β_1_, and α_11_β_1_) predominantly interact with collagen and bind to laminin weakly. Three β_1_ integrins (α_3_β_1_, α_6_β_1_, and α_7_β_1_) and α_6_β_4_ are highly selective laminin-binding receptors (Table 5).

These ECM proteins or the integrin-binding motifs in those proteins have been incorporated on engineered materials to enhance cell attachment. In one study, the cell adherence to glass coverslips presenting different integrin-binding motifs (GTPGPQGIAGQRGVV in collagen I, MNYYSNS in collagen IV, PHSRN in fibronectin, and YIGSR in laminin) differed depending on the motifs, but was much higher than the control case without such motifs [78]. When cells sense these materials that present the peptide motifs, molecular links from cytoskeleton to the materials are enhanced by interactions between integrins and their ligands. Another study explored how the mobility of RGD peptides presented on lipid bilayers influences cell behavior. Two types of substrates with different viscosities (DOPC with a lower viscosity and DPPC with a higher viscosity) were used to change the mobility of the ligands. The average area of immortalized C2C12 mouse myoblast cells increased in response to high ligand mobility that was caused by low viscosity of the substrate. This change was abrogated with the blockade of integrin α_5_β_1_ and α_ν_β_3_, the major receptors for fibronectin [31].

Interactions between materials and cells can cause changes in integrin subunit expression. For example, β_1_ expression levels of two epithelial cell lines, NMuMG and M1 cells, were decreased when grown on soft matrix, while the expression levels of α_2_, α_5_, α_v_, β_3_, and β_4_ were not changed [85]. In another study, poly(L-lactic acid) (PLLA) and PS demixed nanoscale pit textures affected the attachment, spreading, and integrin-mediated focal adhesion structure of human fetal osteoblastic (hFOB) cells. The cells were grown on the PLLA and PS demixed thin materials having circular or channel-shaped pits of three different depths (14, 29, or 45 nm in depth on average) on their surface or flat PLLA materials without a pit as a control. Deeper pits were wider, thereby making the surface areas of deeper pits larger than those of shallow ones (pits of ~14, ~29, and ~45 nm depth had surface areas of 0.09 µm^2^, 0.18 µm^2^, and 0.42 µm^2^ on average, respectively). However, the surface coverage of pits on the thin materials was equivalent to be ~32% of the total surface area for all the three types of materials with pits. Compared with cells on other types of materials including the control case, cells on the materials with 14 nm and 29 nm deep pits showed upregulated expression of α_v_ subunit [86].

For most integrins, ligand binding activity is controlled through conformational rearrangements between the bent conformer having a low affinity and the extended conformer having a high affinity for ligand (Figure 4) [79]. This switchable structures of integrin facilitate adoption of its adhesiveness that is appropriate for the environment [87]. For example, the repetitive mechanical forces applied to bonds between fibronectin coated beads and α_5_β_1_-expressing K562 cells prolonged the bond lifetime, which is linked to a conformational change of integrin to the extended conformer [88]. The structure of integrin as well as its expression level is important to the cellular sensing of substrates.

### 3.2. Sensing Receptors on the Cell Surface—Mechanosensitive Channels and GPCRs

Four types of mechanosensitive ion channels have been found in eukaryotes: cation-selective transient receptor potential (TRP) channels, piezo channels, degenerin/epithelial sodium (DEG/ENaC) channels, and K^+^-selective 2P domain channels [89]. Piezo channels have been identified as the mechanically activated channels that respond to various physical stimuli. In an early study, electrical current was detected in mouse neuroblastoma cells under mechanical stimuli. Expression profiling and RNA interference knockdown of candidate genes demonstrated that Piezo1 was required for coupling of the mechanical stimuli with ion flux. Piezo2 was also characterized as a mechanically activated cation channel through additional experiments [90]. Piezo1 senses physical force through direct transmission by lipid bilayer tension, although the details of the sensing mechanism are still unclear [91]. Piezo proteins sense various physical factors that are important in physiology. Piezo1 channels sense the shear stress generated from blood flow in the vasculature and evoke Ca^2+^ entry into endothelial cells [92]. Piezo2 acts as a rapidly adapting mechanoreceptor for innocuous touch sensation in dorsal root ganglia neurons and Merkel cells by transforming mechanical energy into electrical signals [93].

Another study investigated whether the transient receptor potential cation channel subfamily V member 4 (TRPV4) and Piezo1 channels in chondrocytes can be gated by various physical stimuli. Piezo1 channels exhibited altered responses to tested physical factors, including pressure, indentation, deflection at cell–substrate interface, and membrane stretch, while TRPV4 did not. However, the TRPV4 channel was directly activated and mediated ion currents using only the deflection stimulus applied at cell–substrate contact points. The findings indicated that several mechanosensitive ion channels are not activated by every physical factor, but instead respond to only a specific type of physical factor, as integrin family members do. In addition to Piezo1 and Piezo2 channels, which were previously demonstrated to be general mechanosensors, many unknown mechanically activated ion channels seem to exist [94].

Sensing mediated by mechanically activated ion channels has mainly been studied in prokaryotes. Only a few ion channels have been demonstrated as mechanosensors in eukaryotes [95,96]. To discover additional mechanosensors, small interfering RNA (siRNA) that blocks the expression of piezo proteins was transduced into 25 cell lines. Despite the low levels of Piezo1 and Piezo2 transcripts, MDA-MB-231 cells exhibited a robust transient increase in intracellular Ca^2+^ levels in response to shear stress. Additional tests identified GPR68, a GPCR for sphingosylphosphorylcholine, as another mechanosensor for shear stress that can function separately from Piezo1 channels in MDA-MB-231 cells [97].

GPCRs are the largest group of membrane receptors that cooperate with heterotrimeric guanine nucleotide-binding proteins (G proteins) to mediate a wide range of cell functions. The mediation involves the recognition of physicochemical factors in the cell surroundings in addition to soluble ligands. Apart from GPCR activation by ligand binding, several cellular environmental changes, such as fluid-induced shear stress in blood vessels, osmotic changes, and mechanical pressure, lead to a conformational transition of the receptor from an inactive state to an active state. This transition can mediate downstream signal transduction as well [98,99]. To investigate GPCR-mediated sensing of cell surrounding events various engineered materials have been used to reproduce cellular dynamic environment. For example, stimulation of endothelial cells with fluid-induced shear stress can cause ligand-independent conformational changes of bradykinin B2 GPCR into an active form. In the experiment, a 2 mm wide flow microchamber that exposed endothelial cells to fluid shear stress was used to generate stimulation like that in a blood vessel.

Hypotonic stress and membrane fluidizing agents also have the same effect. These results provided evidence that the GPCR-mediated sensing of physical stimuli is significantly affected by plasma membrane tension or fluidity [98]. The AT_1_ angiotensin II receptor can be activated by the stimulation of human embryonic kidney (HEK) 293 cells by membrane stretch using the patch-clamp technique [100]. GPR68, which was mentioned as a shear stress sensor, can also sense pH changes in the surroundings. At weak alkaline pH, GPR68 is in an inactive state by hydrogen bonding involving histidine. Protonation can disrupt the hydrogen bonding and cause the consequent conformational switch of GPR68 to be in the active state [99].

In this section, we have summarized how cells sense various physical characteristics of engineered materials (Table 6). The sensing process involves folding changes and actions of integrins, mechanosensitive ion channels, and GPCRs.

## 4. Cytoplasmic Mechanotransduction

Mechanotransduction from the cell membrane to the nuclear genome has attracted considerable attention since its first demonstration in 1997 [101]. Mechanotransduction is a process by which cells sense mechanical stimuli and respond to them by conversion of mechanical stimuli to biochemical signals, ultimately leading to specific cellular responses that involve changes in gene expression. Mechanical stimuli can change the functions, growth, migration, and differentiation of cells.

Electrical or chemical signals initiated by extracellular stimuli are transmitted into cells through the cooperation between signaling molecules that activate or localize other proteins. This transmission often involves actions of cytoskeletal proteins, which provide intracellular physical connections. During integrin-mediated sensing, integrins adhere to the outer substrate using their avid ligand binding affinity [80]. Integrin–substrate binding promotes the formation of focal adhesion complex with recruitment of signaling molecules and cytoskeletal proteins around the cytoplasmic tail of integrin [102]. Beginning with the autophosphorylation of a tyrosine residue in FAK, various focal adhesion proteins, including Src family kinases, integrin linked kinase (ILK), and paxillin, can be activated sequentially through phosphorylation [20,103].

Physical features of cell surroundings influence integrin-mediated mechanotransduction and change the relevant cell behavior. For example, mammary epithelial cells (MECs) within ribose-stiffened collagen gels (≥150 Pa) had elevated β_1_ integrin and FAK colocalization and enhanced phosphoinositide 3-kinase (PI3K) signaling, which promotes breast tumor invasion [104]. Nanopatterned surfaces with RGD-functionalized gold dots at regularly spaced intervals also exert effects on the formation of focal adhesions in cells growing on the substrates. Cells cultured on closely arrayed RGD nanopatterns showed elevated levels of vinculin and paxillin throughout the ventral surface of the cells [4]. The activated proteins affect multiple downstream signal cascades involved in a wide range of cellular processes [105]. For example, active Src kinases activate the Ras-Erk pathway, leading to cell proliferation and differentiation. Paxillin and p130Cas, which are also activated by Src kinase, lead to Rac activation, which promotes cell motility [105,106]. Cell survival is regulated by ILK-mediated Akt activity and maintenance of cell polarity requires Cdc42 activity [105].

While soluble factors may trigger cellular signaling pathway independent of the cytoskeletal structure, transduction of physical signals from extracellular substrates into cells requires intact connection between integrin and the actin cytoskeleton. Upon ECM-mediated integrin aggregation, tensin1 molecules are recruited to the focal adhesion site and link between integrins and the actin cytoskeleton [107]. The integrin–ligand interaction induces recruitment of structural proteins, such as vinculin and talin that directly link ECM-bound integrin to actin structure along with tensin1. At the same time, adaptor proteins, including the ezrin-radixin-moesin (ERM) family proteins, Kindlin2, and α-actinin, are also recruited and stabilize the cytoskeletal structure [108,109]. Both signaling molecules and cytoplasmic structural modules are systemically intertwined with each other and cooperatively process the signals sensed by mechanosensors, ultimately eliciting the specific cell behavior. The expression level or activity of each protein involved in the integrin-mediated signal transduction varies responding to the signal from the specific physical stimulus. Altered activities of one or more components in response to the specific physical stimulus affect the overall intracellular signaling network and lead to altered cell behaviors, functions, and fates.

For example, β_3_ integrin and the ERM family, which act as an integrin adapter protein and signaling molecules, mediated transduction of the information of 3D biomimetic microchips into human kidney podocytes. The chips have several protruding channels and were fabricated by photolithography on 22 mm × 22 mm microscope cover glass slides to mimic the natural shape of podocytes. Either unpatterned glass or square-shaped substrate was used as a control. Blocking β_3_ integrin reduced the focal adhesion area in micropatterned cells. Additional supporting data indicated that β_3_ integrin and the ERM family regulate subcellular localization of proteins needed for shape information transduction, thereby maintaining physiologically relevant phenotype of the cells [110].

In the study mentioned above [86], the levels of expressed integrin α_ν_, paxillin, FAK, and autophosphorylated FAK were higher in human fetal osteoblastic (hFOB) cells cultured on nanoscale substrate with topographies harboring 14 nm and 29 nm deep pits, relative to those cultured on substrate with 45 nm deep pits or on flat substrate. The integrin α_ν_-mediated sensing of nanoscale topographies and the subsequently developed focal adhesion structures by highly activated signaling molecules seem to be responsible for hFOB cell attachment and spreading [86]. A study that examined the effects of substrate stiffness on mouse embryonic fibroblast cells found that a rigid substrate was able to activate vinculin by promoting the interaction between vinculin and vinexin α, and could localize vinculin to adhesion sites. High intracellular tension generated when cells were grown on a rigid substrate enabled both activation and localization of vinculin [111].

Mechanotransduction mediated by GPCRs involves two pathways. One is via inositol trisphosphate (IP_3_) and diacylglycerol (DAG) formation. The other is via adenosine 3′,5′-cyclic monophosphate (cAMP) formation. When G_q/11_-coupled receptors sense physical stimuli, phospholipase C (PLC) is activated and cleaves phosphatidylinositol 4,5-bisphosphate (PIP_2_) to IP_3_ and diacylglycerol (DAG) [100,112]. G_S_-coupled receptors activate the cAMP-forming pathway. For example, GPR4, which senses changes in pH, functions in the G_s_-coupled receptor-mediated pathway and GPR68 acts as a G_q/11_-coupled receptor [97,99].

Mechanical stimuli generated by engineered materials are transmitted into cells by signaling molecules and cytoplasmic proteins (Table 7). This mechanotransduction induces changes in expression levels or activities of multiple proteins and, in turn, leads to alteration of cellular processes. Currently, various studies try to find the proteins affected by specific mechanical stimuli and corresponding results in cell physiology, but the relevant detailed molecular mechanisms are still unavailable.

## 5. Nuclear Mechanotransduction

The nucleus is a separate organelle from the cytoplasm that is bound by lipid membranes. The nucleus controls many activities of the cell by regulating the expression of genes encoded by nuclear DNA. During cellular sensing of external cues, cytoplasmic structural proteins and regulatory molecules serve as connectors that link the cell surface and nucleus physically and biochemically (Table 8) [101,113,114].

Linker of nucleoskeleton and cytoskeleton (LINC) complexes, as the name indicates, forms a physical connection within cells [115]. These complexes act as bridges across the perinuclear space by coupling Klarsicht/ANC-1/Syne homoloy (KASH) family members, which are outer nuclear membrane proteins, and Sad1p and UNC-84 (SUN) family members, which are inner nuclear membrane proteins [116]. The large cytoplasmic domains of the KASH proteins interact with cytoskeletal elements including actin filaments, intermediate filaments, and microtubules. Exposed residues of KASH proteins at the perinuclear space bind to the SUN domain at the C-termini of SUN proteins, which are anchored in the inner nuclear membrane. SUN proteins expose their N-termini to the nucleoplasm, thereby binding to the nuclear lamins, which are the major structural units supporting the nuclear membrane.

On the other hand, in the signaling pathway for nuclear mechanotransduction, the action of Yes-associated protein/Transcriptional coactivator (YAP/TAZ) complex is important [114]. The YAP/TAZ complex provides bidirectional communication that mediates cellular mechanoresponses. A study demonstrated that osteogenic differentiation of MSCs on stiff ECM was inhibited by depletion of YAP/TAZ complex [113]. YAP/TAZ-mediated regulation requires Rho GTPase activity and tension of the actin cytoskeleton. Thus, the YAP/TAZ complex can mediate the regulation of cell behavior in response to mechanical cues. In another example, LINC complex-mediated cellular responses to mechanical cues can be induced when the signal molecules phosphorylate several structural proteins in the nuclear mechanotransduction pathway [115]. Accordingly, cooperation between cytoskeletal structure and signaling modules is essential to transmit the stimulus from surrounding environments into the nucleus.

Multiple recent studies have revealed the importance of the translocation of various transcription factors from the cytoplasm to the nucleus and the nonrandom organization of chromosomes in the nucleus for gene regulation [117,118,119]. Mechanical stimuli applied on cell membrane are propagated by various messengers to the nucleus and activate transcription factors. These transcription factors bind to their target genomic sites in the nucleus. However, the mechanism behind the activation of these transcription factors by mechanical stimuli is not completely known. A recent study clarified how the force applied on the cell surface can propagate into the cell nucleus through the cytoskeleton and lamin A/C. The result is the dissociation of intranuclear protein–protein complexes [120]. Functions of lamins ultimately cause the changes in the gene regulation [121,122,123]. In the next section, we review recent studies concerning how external mechanical forces change the states of cellular genomes.

## 6. Changes in Physical and Chemical States of Nuclear Genomes

**Figure 5:**
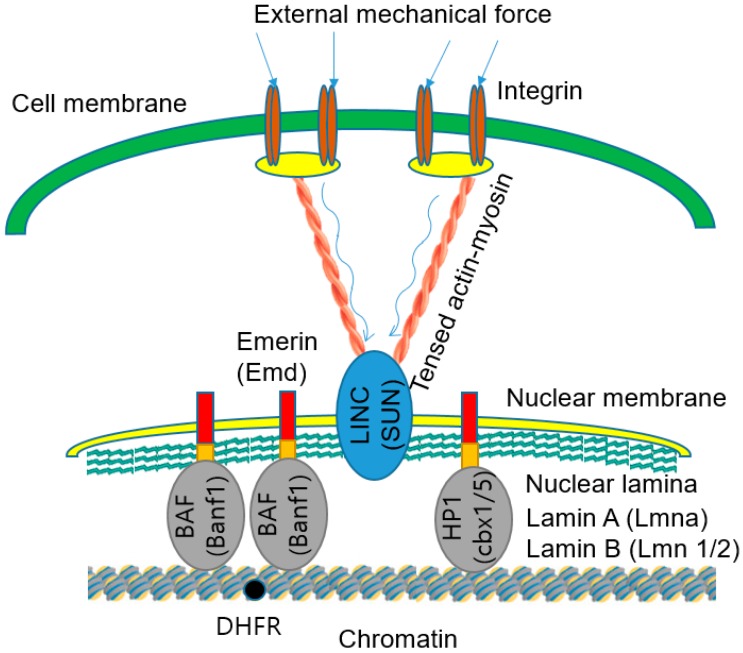
Propagation of external mechanical force from integrins to chromatin [124]. External mechanical forces applied to the cell surface are propagated via integrins and tensed actin-myosin cytoskeleton to LINC complexes and nuclear lamins in the nuclear lamina. The forces are then transferred to the chromatin through heterochromatin protein (HP1), Barrier-to-autointegration factor (BAF) proteins, and other molecules. The forces transferred to the chromatin stretch deform the chromatin segment that contains the DHFR gene. The deformation and stretching of chromatin facilitate binding of transcription factors to the DHFR gene for the upregulation of the DHFR gene transcription. The external mechanical force was provided by ferromagnetic beads attached to integrins in a magnetic field. The magnetic stress was 17.5 Pa.

Figure 5 illustrates how external force applied to the cell surface can affect the chromatin in the nucleus and elevate the DHFR gene transcription [124]. External mechanical stimuli can arrive at the nucleus by mechanical coupling from integrins to cytoskeleton through focal adhesion proteins and enter the nucleus via the LINC complex to nuclear lamin A/B and finally to chromatin.

Gene knockout has been the primary technology that has uncovered the processes of nuclear mechanotransduction. In the approach, the genes encoding the proteins related to mechanotransduction are knocked-out. These genes include Lmnb1/2 (which encodes the elastic lamin B components of the lamina), Lmna (that encodes lamin A, which is important for tissue differentiation and nuclear mechanics), Emd (which encodes the nucleus stiffening emerin protein), cbx1/5 (which encodes HP1), and Banf1 (which encodes BAF) [121,122,125,126]. The knockout of any one of these genes leads to an elevation of spontaneous chromatin movements. The results indicate that HP1 and BAF physically tether chromatin to the nuclear lamins and mediate stress propagation from LINC to chromatins. Reduction of expression for any one of these nuclear proteins leads to the abolition of force propagation and force-induced upregulation of the DHFR gene transcription. These physical links between LINC and chromatins transfer external forces to deform the chromatin structure. Increased stretching of chromatin leads to more pronounced upregulation of the DHFR gene. It is difficult to explain the force-induced upregulation of the DHFR gene by previously suggested models, including translocation or diffusion of FA proteins or other cytoplasmic proteins, such as YAP and TAZ [113] or TWIST1 [30], into the nucleus. Substrate stiffness can regulate Nupr1 expression [127]. To study mechanical force dependent Nupr1 expression changes, tumor-repopulating cells (TRC) were cultured on 2D rigid plastic and 3D soft fibrin matrices. Compared with the 2D surface, the soft 3D fibrin matrices induced reduction of Nupr1 mRNA production and Nupr1 protein levels by approximately 70%. An increase of 3D matrix rigidity and 2D substrate stiffness significantly upregulated Nupr1 expression. These findings indicated that substrate rigidity can regulate Nupr1 gene expression [127].

**Figure 6:**
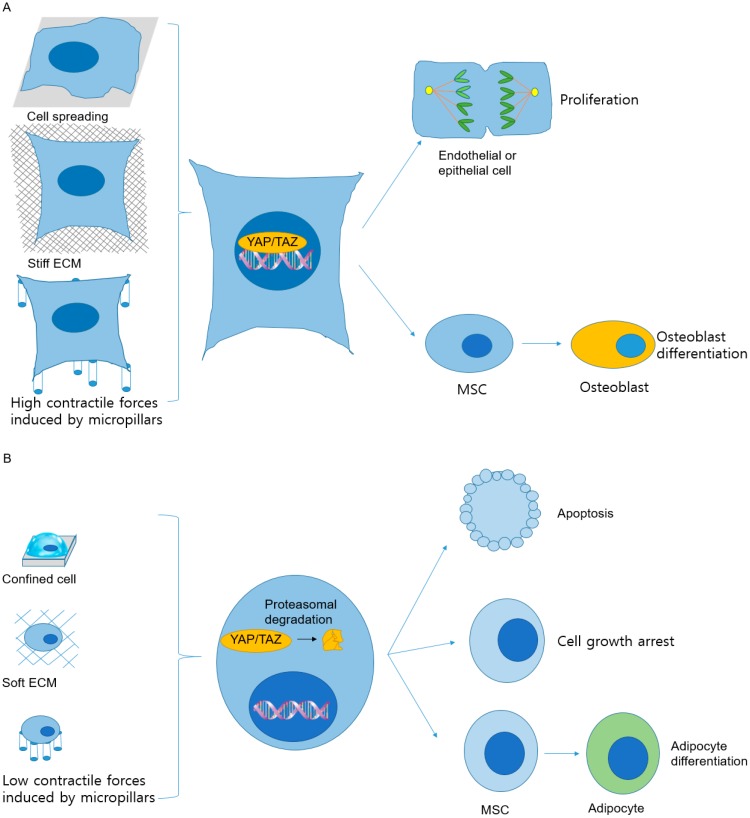
Mechanical stimuli induce YAP/TAZ translocation into the nucleus and determine cell fates [113,147]. (**A**) The translocation of the transcriptional regulators, YAP and TAZ, into the nucleus occurs under mechanical conditions that induce strong intracellular resistive forces and activate YAP and TAZ. In cells spread on an extensive adhesive area, cultured on solid extracellular matrices (ECMs), or stretched between micropillars, YAP and TAZ translocate into the nucleus and become active. Under these conditions, these transcriptional regulators are required for endothelial or epithelial cell proliferation and differentiation of MSCs to osteoblasts. (**B**) The inactivation and relocalization of YAP and TAZ in cytoplasm followed by proteasomal degradation of YAP and TAZ occur when the cells are confined on small adhesive areas or cultured on soft ECMs or on top of micropillars. The degradation results in weak contractile forces. Degradation of YAP and TAZ causes cell apoptosis, growth arrest, or differentiation of MSCs into adipocytes. Furthermore, the degradation and nuclear localization of YAP and TAZ are affected by ECM properties such as stiffness, area, and contractile force.

Figure 6 illustrates how different mechanical conditions affect cell fates through activation and inactivation of the YAP and TAZ transcriptional regulators (Table 9). In the experiment described previously, translocation of most YAP molecules into the nuclei occurred in TRCs cultured on the 2D rigid plastic. The translocation efficiency of YAP into the nuclei in TRCs cultured on soft 3D fibrin matrices was only 18%. Furthermore, many of YAP in TRCs cultured in 3D soft fibrin matrices were phosphorylated at serine 127, and the total amounts of YAP in TRCs on 2D rigid plastic and in 3D soft fibrin matrices were similar. These findings indicate that the phosphorylated YAP could not translocate into the nucleus. Translocation of cytoplasmic YAP into the nucleus can upregulate the expression of the Nupr1, a tumor suppressor. Substrate rigidity could control the fate of stem cells [8].

Cells respond to mechanical cues from their environment, which include the stiffness of the extracellular matrix and interacting forces from neighboring cells [128,129,130]. In tissues of animal and human bodies, stiffness and contractile forces of the local environment surrounding individual cells significantly influence their behaviors [129]. These physical factors induce mechanical stress to initiate mechanotransduction. The stiffness and contractile forces can be also provided by engineered materials. If materials of collagen-coated gels have the stiffness that is similar to that of brain tissue, the MSCs grown on the engineered materials differentiate into neuron cells [8].

Cell shapes are dependent on their microenvironment and are one of the critical regulators for determining the cell growth and states [131,132,133]. Well-spread cells proliferate (Figure 6A), while cells confined in small areas experience apoptosis (Figure 6B). MSCs that are spread out differentiate into osteoblasts (Figure 6A), whereas MSCs restricted in a round shape differentiate to adipocytes (Figure 6B) [134]. The rigidity of the ECM also controls cell functions. Each human organ has a distinct stiffness that is controlled by ECM elasticity and the 3D shape of tissues. MSCs spread on solid ECM differentiate to form osteoblasts (Figure 6A), while MSCs cultured on a soft extracellular matrix differentiate to form neurons and adipocytes (Figure 6B) [8]. The MSCs differentiate into neuron cells, myoblasts, and osteoblasts when cultured on ECM with elasticity ranging from 0.1 kPa to 1 kPa, from 8 kPa to 17 kPa, and from 25 kPa to 40 kPa, respectively. TAZ and YAP are transcriptional mediators that transduce mechanical cues from the microenvironment, and finally cause the biological changes of cells, varying by ECM elasticity and cell shape [113,135]. TAZ and YAP translocate into the nucleus where they act as transcriptional activators during cell growth when cells are on solid ECMs with elasticity of 40 kPa (Figure 6A). On the other hand, YAP and TAZ that are located outside the nucleus are functionally inactivated in cells seeded on soft ECMs such as 0.7 kPa fibronectin-coated hydrogels (Figure 6B) [113]. Among MSCs cultured on micropatterned ECMs having the same rigidity, the cells that are spread out and have large adhesion areas display active TAZ and YAP (Figure 6A). In contrast, the cells confined to a small area have inactive TAZ and YAP (Figure 6B) [113,135]. In addition, the rigidity of a substrate can regulate histone modifications. Rigid substrates or forces applied through integrins increase the methylation of lysine 9 at histone 3 (H3K9) [136]. These changes in histone alter the epigenetic states of cells, a key to alter gene expression.

## 7. Changes in Gene Expression

**Figure 7:**
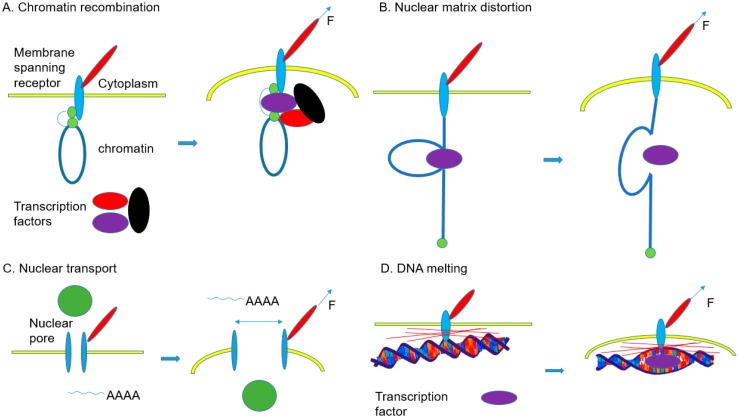
Mechanisms of gene regulation by mechanical stimuli [153]. (**A**) External mechanical forces deform nuclear scaffolds and chromatin organization, thereby altering assembly of transcription factors for gene regulation. (**B**) Forces transferred to specific chromatin regions tethered to lamins or other nuclear membrane receptors regulate the activities of assembled transcription factors or splicing factors. (**C**) Forces applied to nuclear pores change the pore size, thereby altering nuclear transport and gene expression by influencing mRNA transport. (**D**) Forces transferred to DNA through nuclear scaffolds separate the DNA double helix at specific regions, facilitating the binding of transcription factors to the region.

Figure 7 illustrates four mechanisms, in general, by which mechanical forces affect gene expression (Table 10). As shown in Figure 7A, molecules comprising nuclear scaffolds, networks of fibers existing inside of the cell nucleus, might be deformed by external mechanical forces. This deformation leads to an alteration in self-assembly of regulatory protein complexes or other molecule structures related to gene regulation. External mechanical force-induced chromatin structural change leads to differential accessibility for DNA regulatory factors, such as transcription factors and chromatin modifying enzymes. For example, lamin A and emerins bind to transcription factors, and emerins interact with splicing factors. Therefore, forces that propagate through LINC complexes to these molecules can directly affect gene expression by modifying transcription factors and splicing factors [148,149,150]. As shown in Figure 7B, forces applied to a specific region of chromatin that is tethered to nuclear membrane receptors or lamin molecules or internal nuclear scaffold can regulate transcription or splicing factors (violet). For instance, newly synthesized transcripts interact with pre-mRNA splicing machinery. As a result, the forces transferred to these proteins over the matrix attachment region (MAR) regulate mRNA splicing and processing.

Stress or strain transferred to nuclear scaffolds can change higher-order chromatin organization. This conformation change facilitates the access of transcription factors and can influence gene transcription [151]. As shown in Figure 7C, external mechanical forces applied to nuclear pores increase nuclear transport and regulate post-transcriptional process. The external forces transferred to the nucleus alter the size of nuclear pores and change chromatin status, transcription, and mRNA transport through the deformation of the component shapes of pore and alteration of their chemical activities. It is also possible to transfer force to nuclear scaffolds, so the force can directly regulate gene expression [152]. As shown in Figure 7D, mechanical forces applied to specific regions of nuclear scaffolds can stretch certain regions of DNA via MAR tethering. This mechanical force can melt the DNA (especially at AT-rich sites), and the melting of the DNA region facilitates the binding of transcription factors to the region. 

Mechanical forces are important in many cellular processes, such as migration, adhesion, and differentiation, and are also involved in tissue development, regeneration, and morphogenesis. Transfer of external forces to the nucleus through LINC complexes can regulate cell fates during development and differentiation [163]. Higher-order LINC complexes are located at the nuclear boundary. These complexes respond to external mechanical inputs. Mechanical force propagation through nesprin, a key structural protein in the LINC complex, was studied with a mini-Nesprin-2G isoform [164]. The result revealed the involvement of nesprin in force-induced signaling mechanisms. Other proteins are involved in mechanical force propagation. For example, FHOD1, an actin-bundling formin, has both actin- and nesprin-binding domains. It is important in reinforcing LINC complexes during loading of mechanical force [165,166]. The Samp1 inner nuclear membrane protein is also necessary for the formation of transmembrane actin-associated nuclear lines by connecting SUN proteins and nuclear lamina [167].

The nuclear lamina translates these exogenous forces. Lamin A/C deficiency can cause nuclear deformation and defective mechanotransduction under mechanical strain, which can affect mechanically activated gene transcription [168]. The nuclear lamina components can be adjusted by the surrounding environment of cells. In stiffer tissues, the nucleus contains more lamin A/C compared with softer tissues [122]. External force induces a conformation change in lamin [169]. External forces can cause different cytoskeletal prestress, which changes lamin A/C expression, assembly, and disassembly. These events lead to altered gene expression [122]. Lamin A/C is also essential for stem cell differentiation [121,170,171]. Cells in a low-tension environment have more phosphorylated lamin A/Cs, and more lamin A/Cs are degraded in this microenvironment [172,173]. Inner nuclear membrane protein lamin B receptors bind to lamin B and HP1, and LAP2 β binds to lamin B and BAF, resulting in chromatin tethering on nuclear lamina [174,175,176]. This chromatin tethering can increase the stiffness of the nucleus. Osmotic stress also regulates nuclear function and structure [177,178].

Mechanical forces can change the compaction and organization of chromatin. A small deformation of the nucleus leads to an alteration in chromatin remodeling. On the other hand, massive strain causes changes in lamin A/C organization [179]. Chromatin remodeling occurs as an early response to mechanical force. Chromatin condensation in mesenchymal stem cell occurs within 10 min of mechanical stimuli due to the altered activity of the histone modification enzyme [180]. The timescale, nature, and level of mechanical force-induced remodeling of epigenomes depend on the characteristics of the mechanical stimuli. A mechanical force of 1.25 nN applied to the plasma membrane leads to rapid chromatin decompaction [181]. The precise mechanisms for mechanical force-induced epigenomic regulation are still unclear.

In vitro, the activity of histone deacetylases in vascular endothelial cells can be affected by fluid shear stress [182]. Physical stimuli lead to global and locus-specific epigenetic changes related to stem cell pluripotency. For example, in mouse MSCs, oscillatory fluid flow alters the epigenetic state by decreasing DNA methylation of the osteopontin gene promoter and causes the upregulation of the expression of the particular gene [183]. Mouse induced pluripotent stem cells interact with mechanical microenvironment. This interaction increases the levels of H3 acetylation and methylation. These changes improve reprogramming efficiency [184]. Accumulation of mechanical stimuli can result in the phenomenon of ‘mechanical memory’. For example, MSCs cultured on stiff substrates for a long time retain their ‘stiff’ phenotype by localization of RUNX2 and YAP in the nucleus even after these cells are transferred to soft substrates [185].

Mechanotransduction regulates nuclear functions. Mechanical forces have significant impacts on chromatin stretching and gene regulation. Living cells interact with their ECM through many adhesion sites, and the interaction of cell and microenvironment can cause chromatin stretching, compression, or shearing to repress or activate transcription.

## 8. Conclusions

Numerous studies have successfully engineered materials with the specific characteristics that can alter the cellular processes ultimately affecting the various behaviors of the cells. The studies have clarified the transduction pathways of the mechanical stimuli generated by the materials. These scientific efforts have revealed a great deal about the mechanisms for cellular sensing of mechanical cues, transfer of the sensing information to nucleus, and resulting changes in gene expression, and subsequent alterations in cell physiology, function, and fates.

Better understanding of the interactions between materials and cells will enable rational design of engineered materials that allow sophisticated control of cells. In particular, more information is needed to understand how cells distinguish the different characteristics of engineered materials, transfer the specific sensing information to their nuclei, and finally alter their gene expression profiles to respond or adapt to the given artificial environments.

To date, various studies have been conducted to systematically control cellular states that eventually cause adhesion, proliferation, and differentiation of the cells. Diverse approaches to uncover the controlling effects of engineered materials on cellular processes will eventually advance cell-based biomedical applications by adding new physical and chemical tools to the conventional sets of biological cell-tuning tools.

## Figures and Tables

**Table 1:** Studies reporting the effects of substrate stiffness and viscosity on cells.

Material/Substrates	Properties	Cell Type	Effects	Ref.
PAAm gel coated with fibronectin	Stiffness: ~8 kPa and ~100 kPa	NIH3T3 fibroblast cells	The mobilities of the structural proteins are directly influenced by the stiffness of the substrate. The turnover rates of talin, vinculin and tensin1 decreased with increasing ECM stiffness.	[20]
PAAm gel coated with collagen	Stiffness: 150 Pa and 5700 Pa	Human MCF10A and mouse Eph4Ras cells	High matrix stiffness promotes nuclear translocation of TWIST1.	[30]
PDMS micropatterned with laminin	Stiffness: 5 kPa (soft) and 1.72 MPa (hard)	PC12 rat adrenal pheochromocytoma cells	Soft PDMS resulted in significant increase in neurite length	[24]
PDMS micropatterned with fibronectin	Stiffness: 5 kPa, 50 kPa, 130 kPa, 830 kPa, and 1.72 MPa	C2C12 cells	The number of myotube clusters was increased with softer PDMS substrates (5 kPa)	[24]
Arg-Gly-Asp (RGD)-functionalized lipid bilayers composed of either fluid-DOPC or gel-DPPC deposited on glass substrate	Viscosity: 8.4 ×10^−11^ Pa⋅s⋅m (DOPC) 3.0 × 10^−9^ Pa⋅s⋅m (DPPC)	C2C12 cells	Substrates with low viscosity prevented protein unfolding and increased actin flow	[31]

**Table 2:** Studies reporting the effects of geometrical factors on cells.

Material/Substrates	Properties	Cell Type	Effects	Ref.
Mo_3_Se_3_^−^ SCAC nanowire	Inorganic 1D nanowire of 0.6 nm in diameter	L929 fibroblast cells and MC3T3-E1 osteoblast cells	Significant increase in the proliferation of cells was observed in the presence of 1D nanowires.	[34]
Au nanomaterials coated with bovine serum albumin (BSA)	Nanospheres, nanostars, and nanorods of sizes 40 nm, 70 nm, and 100 nm	hMSCs	Size and shape dependent osteogenic differentiation of cells occurred. Nanospheres (40 nm and 70 nm) and nanorods (70 nm) increased the alkaline phosphate activity (ALP) and calcium deposition of the cells.	[35]
PAAm gel micropatterned with collagen I	Stiffness of PAAm gel: Soft (~1 kPa) and stiff (~7 kPa). Diverse shapes of micropatterns with identical area: Square, triangular, and rectangular	MCF-10A cells	Cell−cell junctions could be impaired as matrix became stiffer and the cell shapes became more elongated by the micropatterns. The cell generated tractions that were increased progressively as the pattern shapes were changed from squares to triangles and rectangles.	[40]
Au islands coated with fibronectin	Geometry: Square (250 μm or 500 μm edge), rectangular (125 × 500 μm), and circular (564 μm in diameter)	Normal rat kidney epithelial cells	Geometries of micropatterns altered the cell proliferation by affecting cytoskeletal tension. High cell proliferation was observed on the edges and corners of the square islands.	[37]
Au substrates coated with fibronectin	Circular shape with different diameters (100, 300, 600, and 3000 nm)	Epidermal stem cells	Nanoscale adhesion geometry determined the fate of epidermal stem cells by changing cell shape and AP-1 transcription activity.	[42]
Poly(trimethylene carbonate)	3D microtopographic cell culture chips with concave and convex spherical structures (250 μm in diameter and 1/125 μm^−1^ as principal curvature)	hMSCs	Cytoskeleton-tension-associated pull force on the concave surface: enhanced the cell attachment and increased its migration speed. Push force on convex surface: caused increases in osteogenic differentiation, lamin-A levels, and nuclear deformation.	[43]
Fibronectin fibers and poly oligo(ethylene glycol methyl ether methacrylate) brushes	Quasi-2D fibrous pattern (Dimension: 250, 550, 800, and 1000 nm width, Density: 22 ± 8% and 60 ± 5%)	HaCaT cells	Nanoscale geometry of the ECM acted as an important regulator for cell adhesion, spreading, and shaping. Nanofibrous structures allowed cell adhesions to develop along one axis.	[44]
Au	3D leaf-like structure (nanospikes)	hMSCs	3D nanostructured architecture promoted MSC alignment and neurogenic differentiation	[45]
PDMS coated with collagen I	Smooth and microgrooved topography (10 μm wide, 10 μm apart, and 5 μm deep) Stiffness: 90 ± 8 kPa (soft) and 1500 ± 110 kPa (hard)	hMSCs	Microgrooved stiff substrate led to high cell viscoelastic properties and expression of α-actin and h1-calponin	[48]
PDMS coated with fibronectin	Nanoscale gratings and pillars: 300 nm, 500 nm, and 1000 nm width and diameter Height: 150 nm, 300 nm, and 560 nm	NHLF cells	Nanoscale gratings and pillars facilitated focal adhesion of cells. Nanogratings oriented focal adhesions and nuclei along the nanograting directions.	[49]
PDMS coated with gelatin	Micropatterned substrate: Height (1.5 μm), Groove width (2, 3, 4, and 5 μm), Ridge width (2, 3, 4, and 5 μm)	An accelerated aging cell model derived from induced pluripotent stem cells (iPSCs)	Substrates with specific micropatterns, such as groove width of 5 μm and ridge width of 5 μm, led to higher cell aging via disruption of the connection between the cytoskeleton and nucleoskeleton and triggering of DNA damage	[50]
Ti surface	Nanotopographic pattern, wettability, and mechanical strength	hGF cells	Ti surfaces with pore diameter (74 nm), surface roughness (41.6 nm), surface area (30.4 μm^2^), and hydrophilicity (65.5°) resulted in enhanced cell attachment, proliferation, and differentiation	[56]

**Table 3:** Studies reporting the effects of electric and magnetic field applied to culturing substrates on cells.

Material/Substrates	Properties	Cell Type	Effects	Ref.
Ppy array on Ti surface	Highly adhesive hydrophobic nanotubes and poorly adhesive hydrophilic nanotips	MSCs	The dynamic switching of nanotube/nanotip induced osteogenic differentiation of the cells	[59]
RGD-grafted Fe_3_O_4_ coated silica	Magnetic field induced variation in RGD tether length and mobility on material surface	hMSCs	Restriction in the mobility of RGD on material surface, caused by magnetic field, resulted in enhanced cell adhesion, spreading, and osteogenic differentiation	[60]

**Table 4:** Studies reporting the effects of surface functionalization of substrates on cells.

Material/Substrates	Properties	Cell Type	Effects	Ref.
PDMS Topography: 500, 800, 1000, 1500, and 3000 nm width parallel grooves (400 nm depth)	Functionalization with Matrigel, laminin-111, laminin-211, gelatin, RGD peptide, fibronectin, collagen I, and collagen IV	hESCs	Myotubes aligned perpendicularly on matrigel-functionalized 800 nm nanogrooved substrate through DAPC-mediated cytoskeleton–ECM linkage	[65]
PDMS Topography: Nanopillars, microwells, and micropillars	Functionalization: Fibronectin mixed with collagen I (FC) and laminin mixed with chondroitin sulfate	Human corneal endothelial cells	Micropillars functionalized with FC had high Na+/K+ ATPase and zonula occludens-1 (ZO-1) expression, resulting in enhanced circularity	[66]
Titanium (Ti)	Functionalization: Allylamine plasma polymer layer (PPAAm)	MG-63 osteoblastic cells	Amino groups promoted focal contact formation, such as vinculin, paxillin, p-FAK	[71]
Au	Functionalization: Self-assembled monolayers of alkanethiols like 1-dodecanethiol [*CH_3_ (hydrophobic)], 11-mercapto-1-undecanol [*OH (neutral and hydrophilic)], 11-mercaptoundecanoic acid [*COOH (negatively charged at pH 7.4)], and 12-amino-1-mercaptododecane [*NH_2_ (positively charged at pH 7.4)] * - functional groups	MC3T3-E1 osteoblast cells	OH- and NH_2_-terminated Au surfaces resulted in the selective binding of α_5_β_1_ and α_V_β_3_ integrin for better focal adhesion composition, osteoblast differentiation, signaling, and mineralization	[72]

**Table 5:** Integrin subunit pairing and binding ligands [76,77,82,83,84]. VCAM: vascular cell adhesion molecule, ICAM: intercellular adhesion molecule, LAP: latency-associated peptide, TGF-β: transforming growth factor beta, vWF: von Willebrand factor.

Integrin Subunit		Ligand
β	α	
β_1_	α_1_	Collagen, Laminin
	α_2_	Collagen, Laminin, Thrombospondin, E-cadherin, Tenascin C
	α_10_	Collagen, Laminin
	α_11_	Collagen
	α_3_	Laminin, Thrombospondin
	α_6_	Laminin
	α_7_	Laminin, Tenascin C
β_4_	α_6_	Laminin, Thrombospondin
β_1_	α_4_	Fibronectin, Thrombospondin, Osteopontin, VCAM-1, ICAM-4
	α_5_	Fibronectin, Osteopontin
	α_8_	Fibronectin, Vitronectin, Osteopontin, Tenascin C
	α_9_	Osteopontin, Tenascin C, VCAM-1
	α_V_	Fibronectin, Osteopontin, LAP TGF-β
β_5_	α_V_	Vitronectin, Osteopontin, LAP TGF-β
β_6_		Fibronectin, Osteopontin, Tenascin C, LAP TGF-β
β_8_		LAP TGF-β
β_3_		Fibrinogen, Fibronectin, vWF, Vitronectin, Thrombospondin, Osteopontin, ICAM-4, Tenascin C
β_3_	α_IIb_	Fibrinogen, Fibronectin, vWF, Vitronectin, Thrombospondin, ICAM-4
β_7_	α_4_	Fibronectin, Osteopontin, VCAM-1,
**Leukocyte-Specific**		
β_7_	α_E_	E-cadherin
β_2_	α_L_	ICAM-4
	α_M_	Fibrinogen, ICAM-4
	α_X_	Fibrinogen, ICAM-4, Collagen
	α_D_	Fibronectin, Vitronectin, Fibrinogen, VCAM-1, ICAM-3

**Table 6:** Receptors sensing various physical stimuli.

Receptors	Main Findings	Ref.
Integrins	Integrin can sense diverse physical characteristics of engineered materials such as topography and viscosity.	[7,31]
	Integrin also sense ECM proteins and specific motifs of those proteins when incorporated on engineered materials.	[31,78]
	Integrin is a heterodimer composed of α and β subunits and each integrin selectively binds to different ligands.	[31,76,77,78,85]
	Ligand binding of integrins is controlled by conformational rearrangement between an inactive bent form and an active extended form.	[79,87,88]
Mechanosensitive channels	Piezo channels have been identified as the channels that sense various physical stimuli through transmission by lipid bilayer tension.	[89,90,91,92,93]
	Piezo channels act on coupling of the mechanical stimuli with ion flux.	[90,92,93]
	Piezo1 channel is activated by various physical stimuli, including pressure, indentation, deflection, and membrane stretch, while TRPV4 is activated only by deflection stimulus.	[94]
GPCRs	Several cellular environmental stimuli such as shear stress, osmotic changes, and mechanical pressure can lead to a conformational change of GPCR from an inactive state to an active state.	[97,98,99]
	Fluid-induced shear stress, hypotonic stress, and fluidizing agents have the same effect on GPCR.	[98]
	Various engineered materials have been used to mimic cellular dynamic environment to investigate GPCR-mediated sensing.	[98,99,100]

**Table 7:** Cytoplasmic transfer of the material-sensing signals.

Signal Transfer	Main Findings	Ref.
Integrin-mediated transfer and roles of signaling molecules	Integrin–substrate binding promotes the formation of focal adhesion complex along with recruitment of signaling molecules that subsequently activate or localize other proteins.	[20,80,102,103]
	Beginning with the autophosphorylation of a tyrosine residue in FAK, various focal adhesion proteins can be activated sequentially through phosphorylation.	[20,102]
	Diverse physical features of cell surroundings such as stiffness and topography lead to changes in localization of focal adhesion proteins.	[4,86,105,106]
Integrin-mediated transfer and roles of cytoskeletal structure	Transduction of physical signals from substrates into cells requires intact connection between integrin and the actin cytoskeleton.	[107]
	The integrin–ligand interaction induces recruitment of structural proteins such as vinculin, talin, and tensin1 and adaptor proteins that stabilize the cytoskeletal structure.	[108,109]
	β_3_ integrin and the ERM family, which acts as integrin adapter protein and signaling molecule, mediate transduction of the information of 3D biomimetic microchips.	[110]
GPCR-mediated signal transfer	Receptors coupling with G_q/11_ proteins activate IP_3_ and DAG formation and G_S_ protein-coupled receptors activate cAMP formation.	[100,112]
	GPR4, which senses changes in pH, acts as a G_S_-coupled receptor and GPR68 acts as a G_q/11_-coupled receptor.	[97,99]

**Table 8:** Sensing information transfer between the cell surface and nucleus.

Main Findings	Ref.
LINC complexes act as bridges across the perinuclear space by coupling KASH family members and SUN family members.	[101,115,116]
The cytoplasmic domains of the KASH proteins interact with cytoskeletal elements and the exposed residues of the KASH proteins bind to the C-termini of SUN proteins.	[116]
N-termini of SUN proteins bind to the nuclear lamins.	[116]
YAP/TAZ complex provides bidirectional biochemical connections.	[113,114,115]
YAP/TAZ-mediated regulation requires Rho GTPase activity and tension of the actin cytoskeleton.	[113]
LINC complex-mediated nuclear mechanotransduction can be induced when the signal molecules phosphorylate several structural proteins.	[115]

**Table 9:** Activation/inactivation of YAP/TAZ affects cellular behaviors.

YAP/TAZ States and the Affected Cellular Behaviors		Main Findings	Ref.
YAP/TAZ activation	Proliferation	YAP and TAZ activity, regulated by mechanical properties of multicellular sheets, controls the proliferative capacity of cells.	[137]
		YAP distribution and cell density/cell adhesion area (NIH 3T3 cells) are correlated.	[135]
		The proliferation of endothelial cells is promoted by disturbed flow that causes the activation of YAP/TAZ.	[138]
		YAP1 is an essential modulator for the proliferation of epidermal stem cell and tissue expansion.	[139]
	Osteoblast differentiation	MSCs differentiation is affected by YAP/TAZ activity, which links to mechanical cues from ECM.	[113]
		Runx2-involved gene transcription, repression of PPARγ-involved gene transcription, and differentiation of MSCs are regulated by TAZ.	[140]
		ECM stiffness-dependent osteogenesis of MSCs is promoted by vinculin and enhanced nuclear localization of TAZ.	[141]
		MSC differentiation is extremely sensitive to tissue level elasticity of ECMs.	[8]
		Shapes of mesenchymal progenitors are regulated by MT1-MMP, which results in nuclear localization of YAP and TAZ.	[142]
		YAP activity-dependent MSCs differentiation is regulated by shear stress of cellular environment.	[143]
YAP/TAZ inactivation	Apoptosis	YAP inactivation, caused by the detachment of MCF10A cells, induces anoikis, a kind of apoptosis.	[144]
	Cell growth arrest	Inactivation of TAZ results in growth arrest of glioma cells.	[145]
		YAP inactivation is involved in cell growth arrest and cell contact inhibition.	[146]
	Adipocyte differentiation	MSCs differentiation is regulated by YAP/TAZ activity responding to mechanical cues from ECM stiffness.	[113]
		Runx2-involved gene transcription, repression of PPARγ-involved gene transcription and differentiation of MSCs are regulated by TAZ.	[140]
		MSC differentiation is extremely sensitive to tissue level elasticity of ECMs.	[8]
		Shapes of mesenchymal progenitors is regulated by MT1-MMP.	[142]
		YAP activity-dependent MSCs differentiation is regulated by the shear stress of the cellular environment.	[143]

**Table 10:** Mechanotransduction causes nuclear changes and regulates gene expression.

Nuclear Changes	Main Findings	Ref.
Chromatin recombination	Micropattern-induced reduction of HDAC3 nuclear localization results in decondensation of chromatin. Gene transcription is regulated by chromatin compaction.	[154]
	Decrease of Emd at the inner membrane of the nucleus by extrinsic biaxial mechanical strain leads to the reduction of H3K9me2,3 on chromatin and rearrangements of chromatin for the regulation of gene expression.	[155]
	There are rearrangements of specific chromosomes containing the genes that are regulated by cell geometries. These rearrangements are caused by physical cues from the patterns of cell culturing substrates.	[156]
	Chromatin deformation by magnetic force-induced local stress on CHO cells upregulates the DHFR expression.	[124]
Nuclear matrix distortion	Enhancement of tissue-specific differentiation by mechanotransduction through nuclear lamin A High level of lamin A of cells on stiff matrix stabilizes the nucleus, lamina, and chromatin, which may affect the epigenetic stability and the extent of DNA breaks. Tissue-specific gene expression is regulated by lamin A levels.	[122]
	Acute perturbations of ECM elasticity results in alterations in the levels of lamin A and DNA damage. Slow degradation of lamin A by low phosphorylation leads to lower DNA damage in contractile cells cultured on stiff ECM.	[157]
	There are synergetic effects of collagen matrix rigidity and retinoids on the differentiation of MSCs to osteoblasts. Retinoic acid receptor transcription factors regulate the expression of lamin A.	[158]
Nuclear transport	A mechanotransduction-induced stretch of nuclear pores leads to increase of YAP nuclear localization on stiff ECM.	[159]
	A mechanotransduction-induced stretch of nuclei during cell spreading caused the release of perinuclear Ca^2+^ and elevation of Ca^2+^ level in the nucleus	[160]
	Translocation of cytosolic phospholipase A2 and elevation of Ca^2+^ by nuclear swelling	[161]
DNA melting	Tethering of destabilized DNA regions on MARs results in melting of the double helix.	[162]
